# Promoter features related to tissue specificity as measured by Shannon entropy

**DOI:** 10.1186/gb-2005-6-4-r33

**Published:** 2005-03-29

**Authors:** Jonathan Schug, Winfried-Paul Schuller, Claudia Kappen, J Michael Salbaum, Maja Bucan, Christian J Stoeckert

**Affiliations:** 1 Center for Bioinformatics, University of Pennsylvania, Philadelphia, PA 19104, USA; 2 Department of Genetics, Cell Biology and Anatomy, University of Nebraska Medical Center, Omaha, NE 68198, USA; 3 Department of Genetics, University of Pennsylvania, Philadelphia, PA 19104, USA

## Abstract

**Background:**

The regulatory mechanisms underlying tissue specificity are a crucial part of the development and maintenance of multicellular organisms. A genome-wide analysis of promoters in the context of gene-expression patterns in tissue surveys provides a means of identifying the general principles for these mechanisms.

**Results:**

We introduce a definition of tissue specificity based on Shannon entropy to rank human genes according to their overall tissue specificity and by their specificity to particular tissues. We apply our definition to microarray-based and expressed sequence tag (EST)-based expression data for human genes and use similar data for mouse genes to validate our results. We show that most genes show statistically significant tissue-dependent variations in expression level. We find that the most tissue-specific genes typically have a TATA box, no CpG island, and often code for extracellular proteins. As expected, CpG islands are found in most of the least tissue-specific genes, which often code for proteins located in the nucleus or mitochondrion. The class of genes with no CpG island or TATA box are the most common mid-specificity genes and commonly code for proteins located in a membrane. Sp1 was found to be a weak indicator of less-specific expression. YY1 binding sites, either as initiators or as downstream sites, were strongly associated with the least-specific genes.

**Conclusions:**

We have begun to understand the components of promoters that distinguish tissue-specific from ubiquitous genes, to identify associations that can predict the broad class of gene expression from sequence data alone.

## Abstract 2

A genome-wide analysis of promoters was carried out in the context of gene expression patterns in tissue surveys using human microarray and EST-based expression data. The study revealed that most genes show statistically significant tissue-dependent variations of expression level and identified components of promoters that distinguish tissue-specific from ubiquitous genes.

## Background

The development of an adult from the single cell of a fertilized egg requires a complex orchestration of genes to be expressed at the right time, place, and level. Basic cellular functions require the expression of certain genes in all cells and tissues (that is, in a ubiquitous manner) while specialized functions require restricted expression of other genes in a single or small number of cells and tissues (that is, tissue specific). Both types of genes may be needed for embryonic development as well as for the function of adult cells and tissues. While the details of regulatory mechanisms will vary for individual genes, general features of promoters (and here we will restrict our focus to RNA polymerase II (Pol II) promoters) are likely to facilitate whether a gene will be expressed widely or in a restricted manner. For example, based on the limited number of genes available at the time of the analysis, promoters with CpG islands have been associated with housekeeping genes [1,2]. It is desirable to re-examine this finding in the context of complete genomes for human and mouse and to place it in context with subsequent findings such as the association of CpG islands with embryonic expression [3].

Furthermore, it would also be informative to examine the relationship of CpG islands to the base composition of promoters, and the distribution of motifs thought to be bound by factors closely involved with (or part of) the basal transcription complex. The distribution of major components of the core promoter, the TATA box (TBP/TFIID binding site) and initiator element (Pol II binding site, Inr) [4], and proximal elements such as Yin-Yang 1 (YY1) site [5-8], among genes is not yet well understood. In addition, the functional correlations with tissue specificity and promoter structure are largely unknown beyond the CpG island association. Our goal is to place these components together in general models for tissue specificity using genome-wide surveys of expression in many tissues.

Investigators have searched for combinations of transcription-factor-binding sites that confer tissue-specific expression on particular cell types such as muscle [9] or liver [10] in mammals, or in body plan specification in the fruit fly [11,12] (see [13] for a review). In support of these efforts, analyses of genome-wide expression data have largely focused on identifying common patterns for particular tissues, disease states or signaling inputs. For microarray data, investigators have begun defining these patterns, largely through the application of clustering algorithms [14,15]. Our approach is to rank genes in the spectrum of tissue specificity that runs from expression restricted to one tissue to uniform ubiquitous expression. We can study in detail the distribution of human and mouse genes across the spectrum of tissue specificity and use this to identify commonalities and differences in their promoters with the available complete genome sequences [16], libraries enriched for full-length cDNAs [17-19] and genome-wide surveys of gene expression using microarrays [14,20-24], SAGE [25], mRNAs [18] and expressed sequence tags (ESTs) [26]. We validate patterns discovered in human sequence and expression data by comparison to similar mouse data.

Measures have been developed for overall tissue specificity [3,27,28] that amount to counting the number of tissues that express a gene. These are really measuring tissue restriction, as they do not consider any bias in the expression levels across the tissues that express the gene. Most specificity measures for a particular tissue are equivalent to the relative expression in a tissue compared to the total expression in all tissues considered, (see, for example [29]). We assert that overall tissue specificity measures should take into account the levels of expression in different tissues, not just presence and absence, and that specificity measures for particular tissues should consider the distribution of expression among all tissues in addition to the tissue of interest. Such measures would enable the correct identification of genes as specific for a tissue when that tissue is not the primary site of expression but there are only a few other tissues where the gene is expressed.

A metric for characterizing the breadth and uniformity of the expression pattern of a gene that meets our criteria is the Shannon information theoretic measure entropy. Although entropy has been used previously to identify potential drug targets [30,31] by considering the entropy of the variation of expression levels and to cluster microarray data [32], our direct application of entropy to measuring tissue specificity is unique. Entropy (*H*) measures the degree of overall tissue specificity of a gene, but does not indicate whether it is specific to a particular tissue. To quantify categorical tissue specificity, we introduce a new statistic (*Q*) that incorporates overall tissue specificity and relative expression level. We demonstrate that *H *and *Q *are effective metrics for ranking and selecting genes according to tissue specificity and then proceed to use them to investigate promoter features (CpG islands, base composition, transcription factor motifs) that may be used distinguish tissue-specific genes from nonspecific genes. The association of promoter features with a quantitative assessment of tissue specificity using *H *and *Q *is an important step towards developing models for promoter function.

## Results

### Defining tissue specificity

We begin by defining the measurement of two kinds of tissue specificity, 'overall' tissue specificity and 'categorical' tissue specificity. (To avoid confusion we will always use the words 'specificity' and 'specific' to refer to the degree of tissue-restricted expression a gene exhibits and never as a synonym for the word 'particular'.) Overall tissue specificity ranks a gene according to the degree to which its expression pattern differs from ubiquitous uniform expression. We use the term 'ubiquitous' expression to mean expression at any level above background in all tissues. Categorical tissue specificity places special emphasis on a particular tissue of interest and ranks a gene according to the degree to which its expression pattern is skewed toward expression in only that particular tissue. In both cases, a gene's specificity to a tissue, cell type or other condition is decreased as the gene is more uniformly expressed in a wider variety of conditions. In addition, the categorical tissue specificity should decrease as the tissue of interest becomes a smaller component of the overall expression pattern of the gene.

Given a static multi-tissue expression profile for a gene, there are at least two dimensions along which we can assess the profile to measure tissue specificity. The first dimension is the number of tissues that express the gene above some background level. It can be argued that this dimension measures tissue restriction, that is, a gene shows restricted expression if it is expressed in only a subset of tissues. The second dimension is the uniformity of expression over all tissues that express the gene. A gene that shows significant non-uniform expression is exhibiting tissue-dependent regulation, in addition to any tissue restriction that may be occurring. We assume that a gene that exhibits no tissue-specific regulation will be expressed at the same level in every tissue. We do not assert that such genes are not regulated, only that they are regulated in a way that is not sensitive to tissue.

The term 'most tissue-specific' will refer to the range of genes that are closer to the extreme of expression in a single tissue than to the extreme of ubiquitous uniform expression. We will refer to genes close to the uniform and ubiquitous end as either 'least tissue-specific' or 'nonspecific' though the latter term may not be strictly true. The range in the middle will be termed 'semi-tissue specific'. The term 'housekeeping' has been applied to genes that are widely expressed and may show little tissue-specific changes in expression level. We can use such genes as an example of genes that will tend to be ubiquitously and uniformly expressed and thus ought to be nonspecific on average. We will use the phrase 'gene sharing' to refer to the situation that occurs when a gene is tissue-specific, and is expressed in a small number of tissues that can be said to share the gene.

### Measuring tissue specificity with entropy

**Figure 1:**
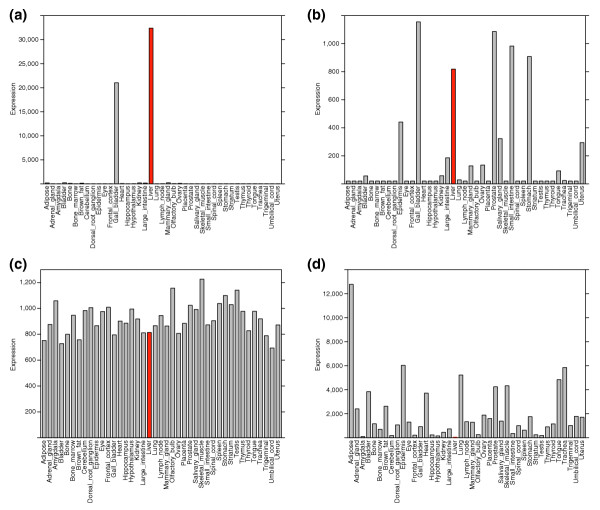
Examples of GNF-GEA expression patterns for mouse genes at selected *H*_*g *_and *Q*. Liver, indicated in red, is the tissue of interest for *Q *values. **(a) **Serum albumin (94777_at *Alb1*) shows very specific liver expression: *H *= 1.3 bits and *Q*_liver _= 2.1 bits. **(b) **For liver-specific bHLH-Zip transcription factor (99452_at *Lisch7*), liver is a strong but not dominant part of the expression pattern: *H *= 3.7 bits and *Q*_liver _= 6.8 bits. **(c) **For chloride channel 7 (104391_s_at *Clcn7*) there is near uniform expression: *H *= 4.3 bits and *Q*_liver _= 10.2 bits. **(d) **Gelsolin (93750_at *Gsn*) is an otherwise widely expressed gene but is expressed at a very low level in the liver: *H *= 4.4 bits and *Q*_liver _= 15.1 bits.

We used two gene-expression datasets to evaluate our methods; Affymetrix-based data from the GNF Gene Expression Atlas (GNF-GEA) [22] and the distribution of source tissues for EST libraries in the clusters and assemblies of ESTs in the DoTS mouse and human gene index [33]. As described in Materials and methods, the GNF-GEA data were used as provided; EST counts in the DoTS gene index were adjusted with pseudocounts and normalized to account for the different number of ESTs sampled from each tissue across all libraries. Given expression levels of a gene in *N *tissues, we defined the relative expression of a gene *g *in a tissue *t *as *p*_*t*|*g *_= *w*_*g*,*t*_/∑_1 ≤ *t *≤ *N*_*w*_*g*,*t *_where *w*_*g*,*t *_is the expression level of the gene in the tissue. The entropy [34] of a gene's expression distribution is *H*_*g *_= ∑_1 ≤ *t *≤ *N *_- *p*_*t*|*g *_log_2_(*p*_*t*|*g*_). *H*_*g *_has units of bits and ranges from zero for genes expressed in a single tissue to log_2_(*N*) for genes expressed uniformly in all tissues considered. The maximum value of *H*_*g *_depends on the number of tissues considered so we will report this number when appropriate. Because we use relative expression the entropy of a gene is not sensitive to the absolute expression levels. To measure categorical tissue specificity we define *Q*_*g*|*t *_= *H*_*g *_- log_2_(*p*_*t*|*g*_). The quantity -log_2_(*p*_*t*|*g*_) also has units of bits and has a minimum of zero that occurs when a gene is expressed in a single tissue and grows unboundedly as the relative expression level drops to zero. Thus *Q*_*g*|*t *_is near its minimum of zero bits when a gene is relatively highly expressed in a small number of tissues including the tissue of interest, and becomes higher as either the number of tissues expressing the gene becomes higher, or as the relative contribution of the tissue to the gene's overall pattern becomes smaller. By itself, the term -log_2_(*p*_*t*|*g*_) is equivalent to *p*_*t*|*g*_. Adding the entropy term serves to favor genes that are not expressed highly in the tissue of interest, but are expressed only in a small number of other tissues. As described earlier, we want to consider such genes as categorically tissue-specific since their expression pattern is very restricted. Figure 1 shows examples of patterns of GNF-GEA expression data for different values of *H*_*g *_and *Q*_*g*|*t*_. The top five genes specific to mouse amygdala, lymph node, and liver as assessed by this data are listed in Table 1. Tables of *H*_*g *_and *Q*_*g*|*t *_values for all genes in all tissues in the GNF-GEA datasets are available in Additional data files 1 and 2.

**Figure 2:**
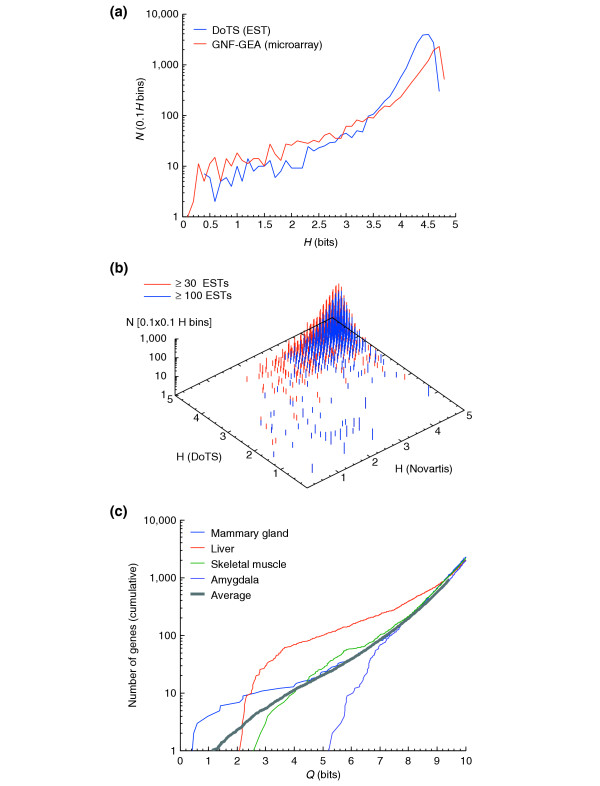
Distributions of *H *and *Q *for different data sources and tissues. **(a) **Distribution of *H *as estimated from GNF-GEA (red line) and DoTS (blue line). The DoTS curve was generated from genes with at least six ESTs. **(b) **Correlation of *H *estimates from GNF-GEA and DoTS. Genes with at least 30 ESTs are shown in red; those with more than 100 ESTs in blue. **(c) **Cumulative distribution of *Q *values for selected mouse tissues and the average for all 39 tissues. Mammary gland, liver, muscle and the amygdala have decreasing numbers of highly tissue-specific genes. Liver has a very large number of relatively specific genes. All distributions peak at 2 log_2_(39) = 10.6 bits and have a tail at high *Q *(not shown) that corresponds to genes that are ubiquitously expressed except in the tissue of interest.

To compare results from microarray and EST-based expression data we mapped the tissues from the GNF-GEA study to the hierarchical controlled vocabulary of anatomical terms used by DoTS and chose a set of 45 tissue terms grouped into 32 groups shown in Table 2. In both cases, the vast majority of genes are widely expressed as measured by *H*_*g *_as shown in Figure 2a. Of the 7,714 probe sets in the GNF-GEA data with an average normalized intensity value above 50 arbitrary units (AU), 6,167 (80%) of genes had *H*_*g *_≥ 4 bits, which implies expression in at least 16 tissues and typically corresponds to wider, but uneven, expression. Only 87 (2%) of genes had *H*_*g *_≤ 1.5 bits, which corresponds to expression in as few as three tissues. Both microarray- and EST-based data yielded similar overall curves. The EST curve peaked at a lower *H*_*g *_than the microarray curve. This was due to the small numbers of EST sequences in some of the tissues we considered; EST counts for tissues ranged from 1,933 in the adrenal gland to 331,582 in the central nervous system (CNS). Genes that are ubiquitously expressed may not have ESTs from several of the lightly sequenced tissues, making them appear to have more restricted expression, and hence a lower entropy, than they really do. Figure 2b shows the correlation between estimates of *H*_*g *_derived from microarray and EST data. Visual inspection of the plot reveals that while there are no strong contradictions between the two methods, quantitative agreement is limited. Detailed analysis shows that the standard deviation of the difference of paired *H*_*g *_values is 0.61 bits. Under the null hypothesis that the estimates from the two data sources are totally uncorrelated the average standard deviation was found to be 0.91 bits. We can reject the null hypothesis (*P *< 10^-5 ^as estimated by Monte Carlo methods). The distribution of *Q*_*g*|*t *_for selected tissues is shown in Figure 2c. These curves can be used to characterize tissues in terms of the number of tissue-specific genes and the amount of gene sharing; for example, liver has a relatively large number of genes shared with a small number of other tissues. In contrast, there were no genes in this set that are uniquely expressed in the amygdala.

It is important to determine how well the *H*_*g *_and *Q*_*g*|*t *_statistics can be estimated from a dataset to determine the smallest meaningful difference in scores and to guide interpretation of gene rankings. To assess the standard deviations of and *H*_*g *_and *Q*_*g*|*t*_, we sampled from the replicates in the GNF-GEA microarray data to compute a large number of *H*_*g *_values for each probe set. We found that the standard deviation for *H*_*g *_was less than 0.2 bits for 97% of genes. *Q*_*g*|*t *_was not estimated as well; the standard deviation was 1 bit or less for 95% of gene and tissue pairs. This was probably due to the high standard deviation of the -log_2_(*p*_*t*|*g*_) term for low expressing gene-tissue pairs. We found much more variation when we measure reproducibility by considering genes that have two or more probe sets (and therefore two or more different transcripts) in the microarray data. In this case, the standard deviation of *H*_*g *_estimates was as high as 1 bit for 97% of the genes but less than 0.3 bits for about 70-80% of the genes. We chose a minimum of 1 bit for *H*_*g *_bins and 2 bits for *Q *bins in the rest of the analyses that require binning. This bin size ensured that most of the genes are in the proper bin and thus the bin could be reliably used to determine associations with the tissue specificity of a class of genes.

### Evaluating a set of housekeeping genes

A test of the *H*_*g *_and *Q*_*g*|*t *_statistics is to determine values for a set of nonspecific genes such as housekeeping genes. A list of 797 human housekeeping genes [35] was evaluated using these statistics based on the GNF-GEA dataset using RefSeq accession numbers to identify appropriate probe sets. The housekeeping genes had a mean *H*_*g *_= 4.6 ± 0.27 bits in a set of 27 tissues with a maximum *H *= lg(27) = 4.75 bits; thus they are nonspecific as expected. Interestingly, a small number of these genes did show some degree of tissue specificity yet were ubiquitously expressed. For example, the median expression of NM_021983 the major histocompatibility complex, class II DR beta 4 gene (32035_at) is approximately 200 AU, but it shows much higher expression in a small set of tissues (spleen, thymus, lung, heart and whole blood), which lowered its entropy. A more extreme case is NM_001502 glycoprotein 2 (zymogen granule membrane protein 2), which is expressed between 250 and 1,000 AU in all tissues except pancreas, where it is expressed at 34,183 AU. This is a ubiquitously expressed gene that entropy categorizes as specific since it showed such extreme tissue-specific induction. The housekeeping genes had a mean *Q*_*g*|*t *_= 9.5 ± 0.14 bits in the same set of tissues. The expected *Q *value for a uniformly and ubiquitously expressed gene is 2 lg(27) = 9.5 bits. Thus, the *H*_*g *_and *Q*_*g*|*t *_statistics successfully captured the expected expression properties of housekeeping genes.

### Most genes are regulated in a tissue-dependent manner

Although the housekeeping genes assessed above have relatively high entropies, they do show some small degree of overall tissue specificity. We therefore sought to determine how many genes show evidence of tissue-dependent regulation. Since random biological and experimental variation introduce fluctuations in the expression levels of genes, we made a probability model of the effect of these fluctuations on the observed entropy. The experimental variability was estimated from the GNF-GEA data using all normal tissues. The random tissue-to-tissue biological variability was modeled by assuming that each gene has an average expression level across all tissues and that the log base 2 of the tissue-dependent fold changes from the average level follow a normal distribution with mean equal to zero and some unknown, but 'small', standard deviation(s). We obtain a conservative estimate of the number of genes showing evidence of tissue-dependent regulation by using *s *= 0.5, which allows for a relatively large amount of variation; up to 1.4-fold tissue-to-tissue variation around the mean expression level in about 63% of tissues and larger changes in the remaining tissues. As a threshold for selecting genes with tissue-dependent expression, we choose *H*_*g *_= 4.52 bits which has a *p*-value of 0.005 under the null hypothesis that all genes are uniform. We then find that 5,837/8,703 (67%) of human genes have entropies less than this and so are probably regulated in a tissue-dependent manner. If we use a more stringent definition of uniform expression that allows half as much variation in tissue-to-tissue expression levels (*s *= 0.25), then the threshold is *H*_*g *_= 4.62 bits and we find that 7,584/8,703 (87%) of human genes show evidence of tissue-dependent regulation. Similar results are found in mouse using all 42 distinct tissues, where the corresponding thresholds are *H*_*g *_= 5.24 bits (*s *= 0.5) and *H*_*g *_= 5.35 bits (*s *= 0.25) and the fractions of genes showing tissue-dependent expression are 5,467/7,913 (69%) and 7,482/7,913 (94%) respectively. Thus we conclude that most genes show evidence of tissue-dependent expression levels.

### Clustering tissues using *Q*

**Figure 3:**
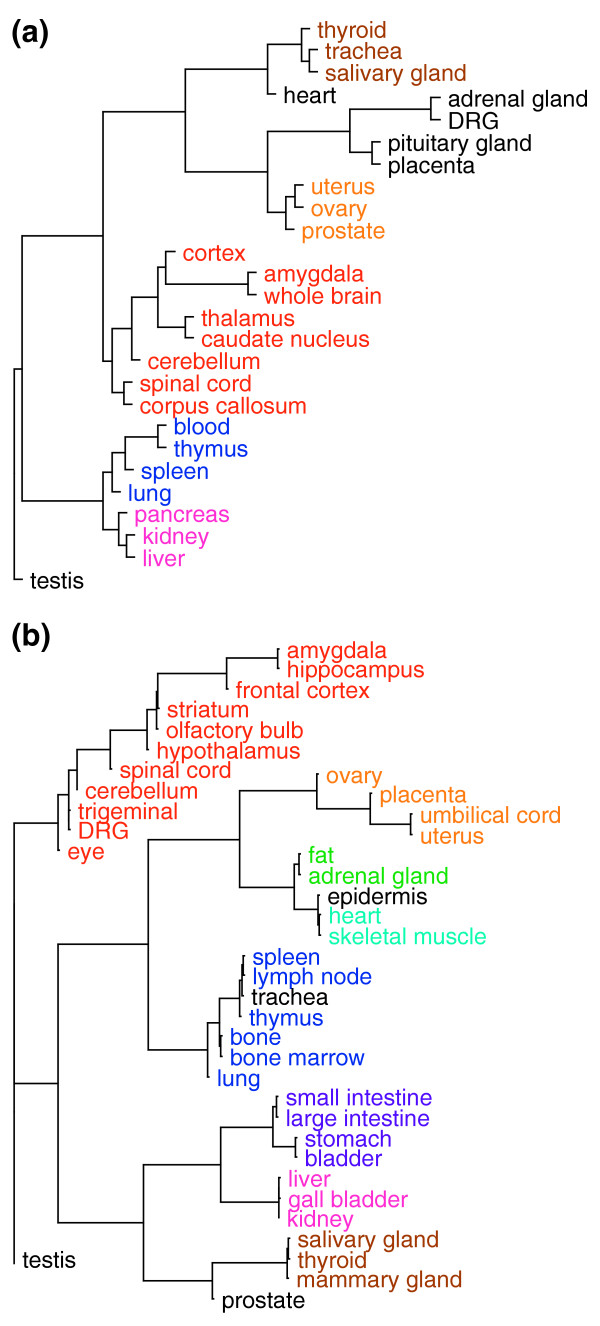
Consensus tissue tree of tissues from human and mouse data. Trees are the consensus of trees created from 5,000 random samples of sets of 1,000 genes from **(a) **3,768 (human) or **(b) **1,786 (mouse) genes with *Q*_*g*|*t *_≤ 7 bits in at least one tissue. The length of the line leading into a node indicates how many trees did not include the set of tissues to the right of the node. The shortest lines correspond to unanimous subgroups. We have highlighted all maximal subgroups that occurred in at least half of the sampled trees. The nervous system is indicated in red, immune system in blue, reproductive tissue in yellow, digestive organs in purple and magenta, muscle tissue in cyan, and glandular tissue in brown. All maximal subgroups that occurred in at least half of the sampled trees. The tissues not included in a highlighted subgroup typically have statistically significant overlap with many of the highlighted tissues as estimated using the hypergeometric distribution.

A test of *Q*_*g*|*t *_with respect to specific genes is to evaluate the tissues in which they rank highly (that is, have low *Q*) for consistency. This was accomplished by clustering tissues with similar tissue-specific genes and inspecting the clusters formed. We used 27 normal human tissues and, separately, 39 tissues from the GNF-GEA data for mouse and selected the genes (*N *= 3,768 human and *N* = 1786 mouse) that express at least 200 AU in at least one tissue and have *Q*_*g*|*t *_= 7 in at least one tissue. With these genes, we made a consensus hierarchical clustering of the tissues as shown in Figure 3. We found that the tissues in the nervous system, reproductive structures (excluding testis), immune system, and digestive system reliably cluster together in both species. In addition, skeletal muscle and heart clustered in mouse; the human survey did not have skeletal muscle. These results suggest that *Q*_*g*|*t *_is correctly identifying tissue-specific genes. Interestingly, testis is an outlier in both trees, indicating that the collection of genes expressed in testis are distinct from any other tissue or organ. Furthermore, *H*_*g *_and *Q*_*g*|*t *_can also be used in conjunction with a tissue hierarchy to answer more complex questions about the tissue distribution of genes such as 'what genes are specific to the brain but are widely expressed throughout the brain?' In Table 3 we list the top five mouse genes expressed specifically but uniformly across three of the highlighted groups in Figure 3b.

### CpG islands are associated with the least tissue-specific genes

**Figure 4:**
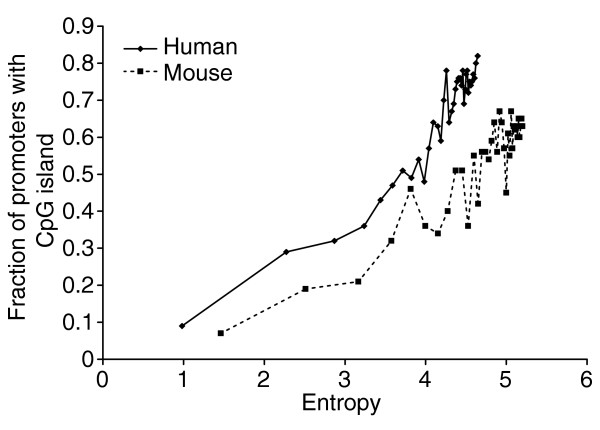
The fraction of start CpG islands in genes ranked by entropy *H*_*g *_increases with entropy. Each point represents the fraction of genes in consecutive groups of 100 genes ranked by entropy *H*_*g *_computed from GNF-GEA data. Genes in this set are expressed above 200 AU in at least one tissue. The human dataset (diamonds) has 26 tissues (maximum *H *= 4.7 bits), the mouse dataset (squares) has 42 tissues (maximum *H *= 5.3 bits).

It has been proposed that CpG islands are predominantly associated with promoters of housekeeping genes [2]. We performed a quantitative test of this hypothesis using the GNF-GEA data and determining the frequency of CpG islands in promoters as a function of *H*_*g*_. We considered only predicted CpG islands that span the start of transcription (see [3] for a justification of this definition), and genes that expressed at least at the median level of 200 AU (that is, were moderately expressed) in at least one tissue, and were represented by a single probe set on the Affymetrix chip used in the GNF-GEA experiments. Promoter sequences were obtained from DBTSS and were based on the 5' ends of full-length transcripts [17]. We found that there is a strong, roughly linear, correlation between a gene's entropy *H*_*g *_and the probability that the gene will have a predicted start CpG island as shown in Figure 4. Start CpG islands were associated with only nine of the 100 most tissue-specific human genes as compared to 80% of the least tissue-specific genes. Similar numbers were found for mouse (7% start CpG island frequency for the 100 most tissue-specific genes; about 64% for the least tissue-specific genes). A comparison of CpG islands from the most and least tissue-specific genes did not reveal any significant difference in the overall base composition, or ratio of observed to expected CpG dinucleotides. The distribution of the position of the 5' end point of CpG islands was also very similar for the most and least tissue-specific genes though CpG islands tend to start further upstream in the least tissue-specific genes (data not shown).

Another group of genes observed to be associated with CpG islands are those expressed in the early embryo [3] from the fertilized egg to the blastocyst. The question arises as to whether there is an association of genes having start CpG islands and the developmental stage of expression (that is, embryonic versus adult) in addition to the one for tissue specificity. We investigated this possibility in the mouse using DoTS [33] EST and mRNA assemblies by tabulating the number of DoTS genes that contain at least two ESTs from a mouse early embryo library as shown in Table 4. We considered 933 genes with start CpG islands (CGI+) and 1,007 genes without start CpG islands (CGI-) that were expressed in the adult. If there were no developmental bias, this distribution of CpG+ and CpG- genes should be maintained in genes expressed in the embryo. However, only 139 (14%) of the CGI- genes were expressed in the early embryo in contrast to 365 (39%) CGI+ genes (*P *= 3 × 10^-70 ^exact binomial). Therefore, a gene expressed in the adult was 2.8 (= 0.39/0.14) times more likely to be expressed in the early embryo if it contained a start CpG island. Furthermore, the most tissue-specific genes expressed in the adult were four times more likely to have been expressed in the early embryo if their promoter contained a start CpG island. These results strongly suggest that CpG islands are promoter features for both embryonic and the least tissue-specific genes.

### Base composition of promoters depends on specificity

**Figure 5:**
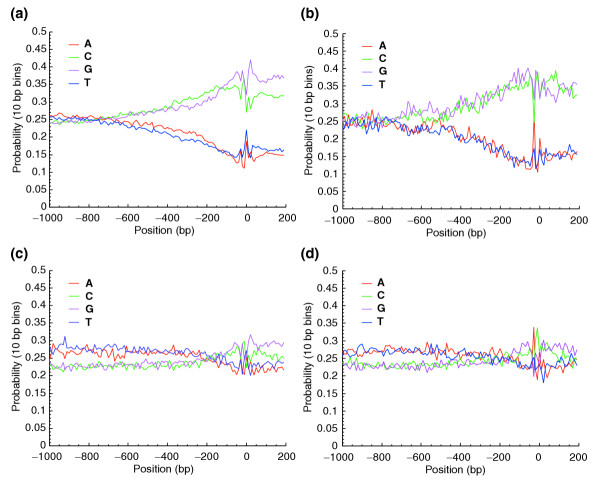
Base-composition profiles for ubiquitous and tissue-specific genes with and without start CpG islands. Data is for human genes; similar patterns were observed in mouse. **(a) **Ubiquitous genes with a CpG island; **(b) **tissue-specific genes with a CpG island; **(c) **ubiquitous genes with no CpG island; and **(d) **tissue-specific genes with no CpG island. Note differences in upstream C+G content, peak sizes at TATA box (-35 bp) and initiator positions, and downstream C versus G differences.

Analysis of base-composition profiles of promoters provides clues to common features, including motifs associated with promoter categories. We examined the base composition profiles of human promoters of high (0 ≤ *H*_*g *_≤ 3.5 bits) and low (4.4 ≤ *H*_*g *_≤ 4.71 bits) tissue-specificity genes. We considered CGI+ and CGI- genes separately, as it is clear the presence of a CpG island will strongly influence the base composition and that the fraction of start CpG islands varies with entropy. In addition, the presence of a start CpG island may indicate a different regulation mechanism related to either tissue specificity or embryonic expression (or both). The number of promoters from DBTSS in these four classes that were used in the analysis were: 310 CGI- and 129 CGI+ high specificity; 342 CGI- and 1,501 CGI+ low specificity. Genes that have only non-start CpG islands represented a minor component and were not included in this analysis. We used the full set of normal tissues in the first GNF-GEA microarray study for human and mouse. Base composition profiles with 10 base-pair (bp) windows are shown in Figure 5 for human genes. Each of the features we report were observed in human and mouse (unless noted otherwise) and compare G to C or A to T over spans of at least 10 positional bins; the probability of observing a feature at least this long by chance is less than 0.5^10 ^which is equivalent to 0.001. Promoters of CGI+ genes (Figure 5a,b) shared features but could also be distinguished on the basis of tissue specificity. A common feature of CGI+ promoters was the increase in C+G content that starts at 1,000 bp upstream of the transcription start site and continues at 200 bp downstream. The C+G bias reached p(C+G) = 0.7 at the start of transcription and continued into the 5' UTR. Nonspecific (Figure 5c) and tissue-specific (Figure 5d) CGI- genes still showed a C+G bias around the start of transcription, but it was much smaller in magnitude at p(C+G) = 0.54. The low specificity CGI+ genes (Figure 5a) showed upstream base composition biases that were not found in any of the other three gene classes. There was a preference for C over G (p(C) > p(G)) in the (-350, -150) region and also a preference for p(A) > p(T) in the -600, -200 region in human (this region is located (-400, -150) in mouse). In tissue-specific CGI+ (Figure 5b) genes the strong C+G bias held but p(C) = p(G), except for the (+50, +100) region where p(C) > p(G). These base-composition differences observed between nonspecific and tissue-specific promoters over regions of hundreds of base-pairs, even in the context of a CpG island, suggest different structural features and regulatory mechanisms for these CGI+ classes.

Most striking were differences between nonspecific and tissue-specific promoters that are independent of the presence of a CpG island. A sharp spike in the proportion of A and T was seen in the (-50,-1) region for all classes but was most pronounced in the tissue-specific promoters (Figure 5b,d). These spikes correspond to the presence of a TATA box and suggest a correlation of this motif with tissue-specific genes (explored more fully later). Conversely, all low-specificity genes (Figure 5a,c) shared a common feature in the (+1, +200) region where p(G) > p(C) and p(T) > p(A) that was not seen in tissue-specific genes (Figure 5b,d). As shown later, this low-specificity feature could be partially explained by the presence of a YY1 motif. These base-composition differences observed between nonspecific and tissue-specific promoters are likely to indicate motifs that distinguish the two classes.

### Selected transcription factor motifs in the core promoter

We next examined the distribution of basic core promoter features: the TATA box, the initiator element, and two binding sites for selected ubiquitous transcription factors, Sp1 and YY1, to see if their presence in the proximal promoter was correlated with the tissue specificity of a gene. Two approaches were taken using different datasets and motif-searching methods that gave similar results, providing independent confirmation of results. First, we searched for core motifs using weight matrix hits in promoters of genes selected using *H*_*g *_calculated from the GNF-GEA data. Second, we searched for core motif consensus sites in promoters of genes selected using *Q*_*g*|*t *_calculated from EST data.

### TATA boxes are associated with tissue-specific genes

We grouped the human genes that expressed at least 200 AU (average value) in the GNF-GEA data by entropy and start CpG island status. The number of genes in each category is shown in Table 5 along with a summary of results. We used alignments of position-specific scoring matrices and scoring thresholds included in the Eukaryotic Promoter Database (EPD) [36] to identify the TATA box and initiator element. Matches to these motifs were preferentially located at the expected positions relative to the transcription start site based on the ratio of the number of observed set to the expected number using a set of random sequences with the same position-dependent base composition as each of the promoters.

We searched for the TATA box in the (-45, -10) region where the average observed/expected ratio for the TATA box was 3.1. As shown in Table 5, the most-specific CGI- genes were six times more likely to have a TATA box than the least-specific CGI+ genes (117/215 (54%) versus 183/2072 (9%), *P *≈ 0 exact binomial). Similar numbers are found in mouse (52%/11% = 4.7) This trend also holds within CGI- genes and CGI+ genes. The most specific CGI- genes were three times more likely to have a TATA box than the least specific CGI- genes (117/215 versus 110/607, *P *≈ 0 exact binomial). While less common in CGI+ genes, TATA boxes were still almost four times as likely to be found in the most specific CGI+ genes than the least specific CGI+ genes (19/56 versus 183/2,072, *P *= 2 × 10^-7 ^exact binomial). Thus TATA boxes are clearly associated with tissue-specific genes and provide a second axis (with CpG islands) for distinguishing between the most and least specific genes.

In contrast, the frequency of occurrences of the initiator element (Pol II binding site) was roughly constant across all tissue-specificity classes for both CGI+ and CGI- genes. We searched for the initiator element in the (-10, +10) region. It occurred in 762 of 1,118 (68%) of CGI- genes and 1,273 of 2,434 (52%) of CGI+ genes. Similarly, it occurred in 149 of 215 (69%) of the most specific genes and 388 of 607 (64%) of CGI+ genes. The observed frequency of TATA+/Inr+ promoters was not significantly different from the expected rate assuming independence of the two individual features (data not shown).

### Sp1-binding sites are weakly associated with the least tissue-specific genes

Sp1 [37,38] is a ubiquitous transcription factor with a G-rich binding site with consensus sequence GGGCGGG that might explain the observed G-richness of the 5' UTR in non-specific genes. We used the GC-box weight matrix and scoring threshold from EPD [36] to identify Sp1 sites. We found that Sp1 sites are preferentially located in the (-150, +1) region in all sets of genes where they occurred on average at twice the expected rate in agreement with previous findings [36]. In both human and mouse, Sp1 sites were rarely found in the 5' UTR despite the G-richness of this region; they occurred at the expected rate of between 2 and 5%. Thus Sp1 sites were not the cause of the G-richness in the 5' UTR.

Sp1 sites are associated with CpG islands but are an important component of GGI- promoters as well. Considering just the (-150, +1) region, Sp1 sites occurred in 1,105/2,434 (45%) of human CGI+ gene promoters, and 316/1,118 (28%) of CGI- genes at about 2.5 to 3.0 times the expected frequency in both cases. Frequencies in mouse are 927/2075 (45%) of CGI+ promoters and 464/1652 (28%) CGI- promoters. Sp1 sites were also weakly associated with the least specific genes occurring in 1,105/2,679 (41%) of these genes as compared to 94/271 (32%) in the most tissue-specific genes (*P *= 0.016). Similar numbers are found in the mouse; 38% of the least specific and 26% of the most specific promoters have Sp1 sites. Thus, although Sp1 shows a preference for the least tissue-specific promoters, it is not a strong predictor of the tissue specificity of a gene.

### YY1 binding sites are associated with low-specificity genes

**Figure 6:**
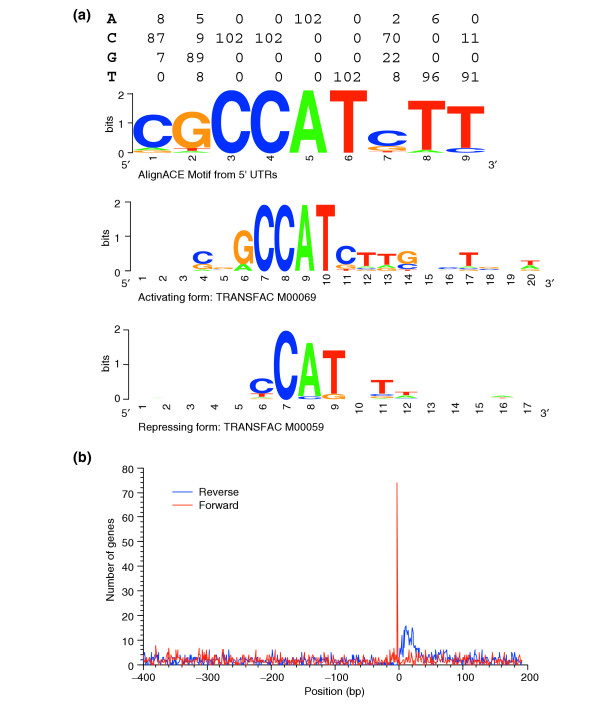
YY1 motifs are found downstream of the transcription start site, depending on their orientation. **(a) **The top image shows a logo [69] representation of the YY1 motif in the (+10, +20) region of human CGI+ promoters identified using AlignACE. It is based on 102 sequences. The other two logos are for weight matrices contained in TRANSFAC v7.3 that represent activating and repressing YY1 binding sites. **(b) **Plot of the positional distribution of predicted YY1 sites and the fraction of genes with a predicted YY1 sites in the (+1, +60) region. YY1 sites were predicted using a weight matrix generated using AlignACE. YY1 sites are more than almost three times (*P *≤ 2 × 10^-7^) as common in genes with nonspecific CGI+ genes (11%; *N *= 2,072) than in CGI- genes (4%; *N *= 607) and occur at more than 10 times the expected rate. Similar trends are observed in genes with 3 ≤ *H *≤ 4 though with lower absolute and relative rates. The difference between CGI+ and CGI- genes is not statistically significant for genes in the 3 ≤ *H *≤ 4 bin. Essentially no YY1 sites where observed in specific genes with *H *≤ 3 bits whether or not they had a CpG island.

The transcription factor YY1 [5-8] is also ubiquitously expressed and is thought to bind close to [39] and downstream of the transcription start site. There is evidence that the function of YY1 depends on its orientation [40]. The location and G-richness of the reverse complement consensus sequence (AANATGGCG) make YY1 a candidate for explaining the prominent G > C feature in the (+1, +200) region of low-specificity genes. We consider YY1 because a YY1-like motif was frequently included among the most statistically significant motifs identified by the motif discovery programs AlignACE [41] and MEME [42] in the (+1, +60) region of nonspecific CGI+ promoters (Figure 6a). Our form is most similar to the activating form [43], which may be associated with low-specificity genes. Because of the demonstrated functional sensitivity to the orientation of binding sites we considered each orientation separately. Indeed, as shown in Figure 6b we found each orientation exhibits different position preferences. Sites in the reverse orientation (YY1_r_) were preferentially located in the (+1, +25) region but with some elevated levels to +80 bp. Start positions of sites in the forward orientation (YY1_f_) showed a very sharp preference for -3 bp, which probably represents a YY1-like initiator sequence reviewed elsewhere [44]. Both orientations were found predominantly in the least specific genes (Table 5). YY1_f _initiator sites are rare; only 55/2,679 (2%) were found above background in human low-specificity genes. The rate in mouse, 22/2,832 (0.8%) of low-specificity promoters, is even lower. The YY1_r _sites are more common and were found above background in 217 (8%) of the 2,679 least specific genes. YY1_r _sites were more common in CGI+ genes than in CGI- genes (202/2,072 (10%) versus 15/607 (2%) *P *= 3.7 × 10^-9 ^two-population binomial). The corresponding rates in mouse confirm these observations; 178/2,832 (6%) for all low-specificity genes and 152/1,779 (9%) in CGI+ and 26/1,053 (2%) of CGI- low-specificity promoters. These YY1-like sites therefore constitute a feature strongly associated with the least specific genes and may partially explain the observed G > C ratio in the (+1, +200) region.

### *Q*-based analysis of core promoter motifs

**Figure 7:**
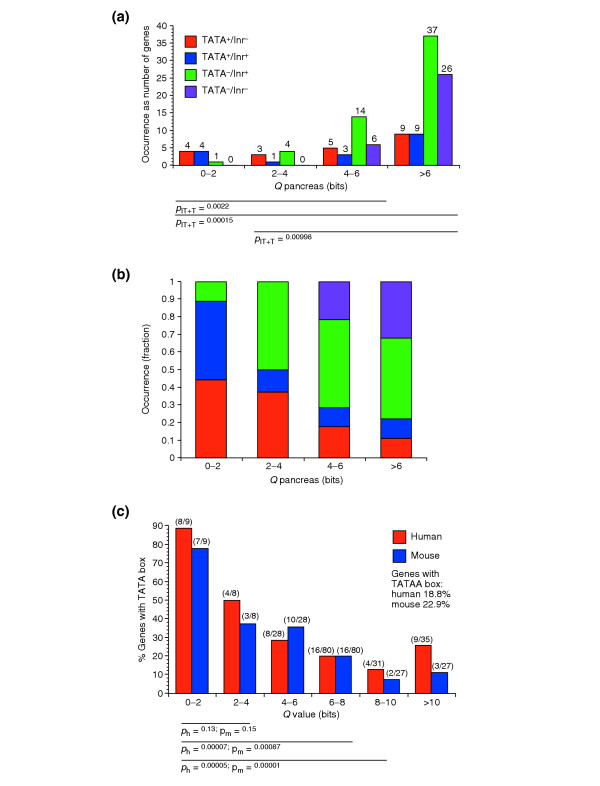
The distribution of TATA box and initiator element (Inr) in pancreas-specific genes. One hundred and sixty pancreas genes were divided into bins according to their *Q*-value. Genes that have a TATA box, an initiator with the motif YYANWYY, both, or none of these two motifs, are shown. **(a) **Absolute numbers of genes with core promoter motifs. Red bars, TATA only; blue bars, TATA and Inr; green bars, Inr only; purple bars, none. The *p*-values for pairwise comparison of distributions (TATA/total) are given below the graph. *P*-values were calculated for the sum of genes with TATA box (with and without initiator). **(b) **Results from (a) plotted as fractions of genes with each motif status within a bin. **(c) **Number of TATA boxes found in orthologous human and mouse gene pairs. Statistical significance of differences between *Q *bins are indicated.

A second analysis of TATA box and Inr motifs was done to determine if the association of the TATA box with tissue-specific genes is also found in genes ranked by *Q *and is robust to using EST data as well as promoters that did not specifically rely on full-length cDNA clones. The definition of *Q *implies that genes with a particular *Q*-value can have a variety of *H*_*g *_values and thus it may be more difficult to identify features related to tissue specificity. We tabulated all DoTS genes that contained at least two ESTs from an islet-cell library then ranked the genes by *Q*_pancreas _computed using EST counts. We used *Q*_pancreas _≤ 7 bits as the criterion for selecting pancreas-specific genes which we grouped into 2-bit Q intervals. For comparison we selected 50 genes with *Q*_pancreas _= 8.5 bits, and 50 genes with 10 ≤ *Q*_pancreas _≤ 10.6 bits. Genes with high specificity for the pancreas (0 ≤ *Q*_pancreas _≤ 2 bits, *N *= 9) preferentially had TATA boxes (8 of 9) with half of these also having an initiation element (4 of 9; Figure 7a). With decreasing specificity, the fraction of genes containing TATA boxes drops with only18 of 81 (2/9) genes with *Q *> 6 bits having TATA boxes. Thus, the strong correlation of TATA boxes with specific genes found with *H*_*g *_and microarray data was also seen with *Q *and EST data for pancreas-expressed genes. Also consistent is the observation that initiator elements were found at similar frequencies (around 60%) across all specificity classes (Figure 7b). Similar patterns were observed in other tissues (data not shown).

The consistency of findings for the TATA box with human islet genes based on *Q *and ESTs was next tested with orthologous genes in mouse. This test provides a measure for whether the global pattern observed (TATA box with tissue-specific genes) is also found for the same set of genes in another mammal. We also added bins of genes with higher *Q*-values that represent more widely expressed genes. For each human gene, the orthologous mouse gene was determined (see Materials and methods for details) and analyzed as described above. Overall, 18.8% of the human genes and 22.9% of the mouse genes that were analyzed carry the TATA box motif. Except for the last group (*Q *>10 bits) the percentage of the genes with TATA box motifs decreases with the increase in the *Q*-value. This is to be expected since genes with high *Q *may be specific to other tissues and hence are more likely to have a TATA box. Discrepancies between human and mouse promoters were noted for only about 10% of all human-mouse pairs analyzed and may reflect sequence differences and possible annotation discrepancies for the transcription start site. Nevertheless, there is overall excellent agreement for the presence of TATA motifs in human and mouse genes. Thus, our assessment of preferential presence of transcription regulatory motifs in the human pancreas-expressed genes also applies to their mouse orthologs. We conclude that genes expressed with restricted tissue-distribution may be preferentially regulated via TATA-mediated transcription, and that genes with broader expression profiles are more likely to be regulated by non-TATA mediated mechanisms (such as YY1).

### Promoter classes

Since the presence or absence of a start CpG island and a TATA box appear to be the primary sequence feature that correlate with tissue specificity, we consider them in more detail. We observe that CpG islands and TATA boxes are not mutually exclusive features of promoters and so we consider all possible combinations of these features.

### Frequency of promoter classes

**Figure 8:**
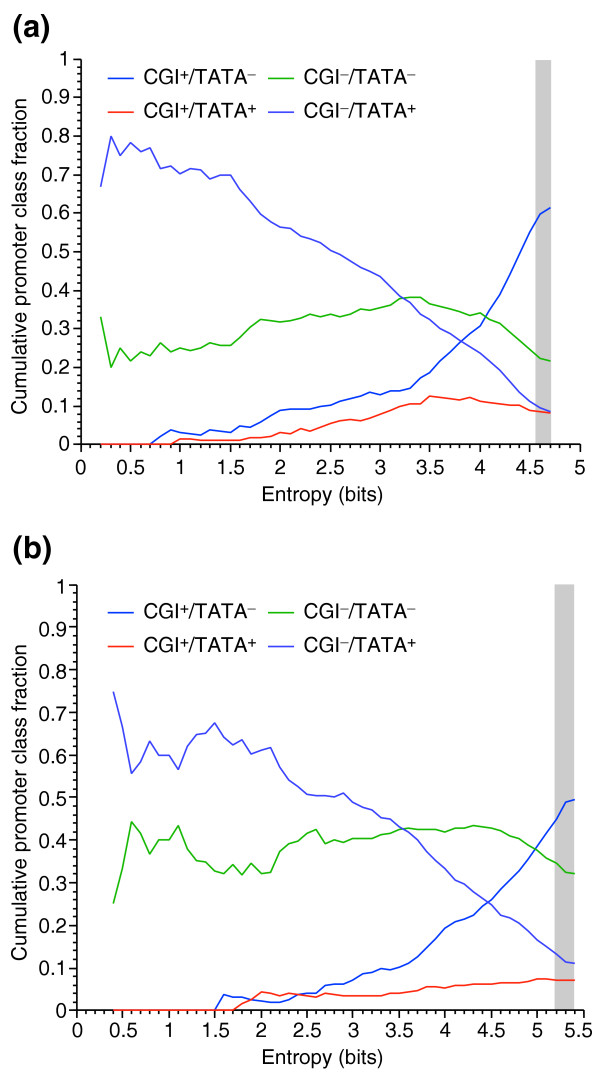
The cumulative distribution of promoter classes as a function of entropy is similar in human and mouse. The cumulative fractions of genes with all possible combinations of CGI and TATA box features for **(a) **human and **(b) **mouse as a function of entropy *H*_*g *_as computed from GNF-GEA data is shown. For example, in human about 50% of the genes with *H*_*g *_≤ 2.5 have a CGI-/TATA+ promoter. The gray bars indicate the entropy range that is not significantly different from uniform ubiquitous expression. Curves are compiled from genes that express above 200 AU in at least one tissue. As expected, CGI+/TATA- genes are most common in less specific genes and CGI-/TATA+ genes are most common in tissue-specific genes. CGI-/TATA- genes are very common and are found nearly uniformly at every level of specificity. Furthermore, CGI+/TATA- and CGI-/TATA+ genes are both common in mid-specificity (3 ≤ *H*_*g *_≤ 4) genes showing that specificity is not determined by these features alone. The trends in human and mouse data are nearly identical despite the lower rate of CpG islands in mouse. The large variations in the graph at low entropy are due to the noise inherent in the small number of genes in this range.

Figure 8 shows the cumulative fraction of each class of promoter as a function of increasing *H*_*g *_in human (Figure 8a) and mouse (Figure 8b). The data from human and mouse follow similar trends even though the mouse has a lower proportion of CGI+ genes. Overall, CGI+/TATA- genes are the most common, at 50-60% depending on the species. Interestingly, the CGI-/TATA- class is the second most common overall, comprising 20-30% of genes, depending on the species. Genes in this promoter class are roughly equally common across the entire entropy range and are the most common promoters in the mid-specificity range in both species. The classes CGI-/TATA+ and CGI+/TATA+ are the least common (8 to 12% overall). CGI-/TATA+ genes are concentrated in the most specific genes. CGI+/TATA+ are found relatively uniformly across all but the most specific genes. Although the TATA box and CpG islands are strongly predictive of a gene's entropy, Figure 8 also illustrates the limitations of the promoter classes as an explanation for expression patterns. First, although the CGI-/TATA+ and CGI+/TATA- classes are strongly associated with the most and least tissue-specific genes (respectively), instances of genes in each class cover virtually the entire range of tissue specificities. Second, the CGI-/TATA- class is the second most common, illustrating that any degree of tissue specificity can be obtained without these sequence features.

### Functional assessment of promoter classes using Gene Ontology terms

To try to understand the functional correlates of the four promoter classes, we looked for trends in the cellular localization and biological process of the products of genes from each promoter class. We used the DAVID system [45,46], which identifies over-represented Gene Ontology (GO) [47] terms in a set of genes. A summary of the results for human and mouse genes are shown in Table 6. In each case the set of genes in each promoter class were compared to all genes on the corresponding Affymetrix chip.

Products of genes in the CGI-/TATA+ class were often (70/198) located extracellularly. Examples of such genes are the insulin-like growth factor family, serum albumin and chymotrypsin. Some extracellular CGI-/TATA+ genes, such as luteinizing hormone beta (LHB) and bone morphogenetic protein 10 (Bmp10) in the mouse, have a high *H*_*g *_because they are not induced in the tissues or at the developmental stages surveyed, but otherwise fit the pattern of secreted proteins. Gene products that are secreted from the cell must be produced at high level to be effective. Indeed we found the maximum expression level of TATA+ genes is higher than TATA- genes; 454/745 (61%) of TATA+ genes express at least 1,000 AU in one or more tissues, whereas only 1,321/3,773 (35%) of TATA- genes express that highly (*p*-value = 0; two-sample binomial population). A second group of CGI-/TATA+ that is common, but with a *p*-value just over the *p*-value cutoff are the muscle contraction-related genes, actin, troponin and members of the myosin family. Products of these genes are also required in large amounts to create the contractile apparatus but are only produced in a few cell types. The biological processes that are enriched in the CGI-/TATA+ class differ between human and mouse, but nearly all of them are descendants of the GO term 'response to stimulus' (GO:0050896).

The CGI+/TATA- promoters produce proteins that are typically located in the cell, especially in the cytoplasm and mitochondrion. These locations are consistent with many housekeeping functions. The human results for biological process suggests a large number of housekeeping processes, but these were not confirmed in the mouse using all CGI+/TATA- genes. When we consider just the least specific CGI+/TATA- mouse genes (4.45 ≤ *H*_*g *_≤ 5.57 bits), we find cellular locations (including the nucleus) and biological processes that match the human results.

No significant concentrations of cellular locations or biological processes were found among the CGI+/TATA+ genes. A manual examination of genes in both human and mouse identifies a number of heat-shock proteins, histones and ribosomal proteins although these are not statistically significant as a result of the multiple testing correction. Many of these genes fit the expected expression pattern in that they are widely expressed and at high levels.

Interestingly, the products of CGI-/TATA- genes are often located in the plasma membrane (244/499 of human genes with a cellular location) and support signaling and response to the environment. Such products, for example, bradykinin receptor B2, prolactin receptor or protocadherin 9, may be expressed in a tissue-specific pattern, but not at the high levels required for secreted proteins. The exact biological process GO terms that are statistically significant vary between mouse and human, but a common core includes defense response (GO:0006952), immune response (GO:0006955) and response to stimulus (GO:0050896). Thus these genes are similar to CGI-/TATA+ genes in that they are involved in response, but are not (typically) required to be expressed at such high levels.

## Discussion

We have applied Shannon entropy as a novel measure of overall tissue specificity of gene expression and have created a new statistic *Q *to assess the categorical specificity of a gene for a particular tissue. We have evaluated the performance of entropy on microarray-and EST-based estimates of tissue-specific expression and found that it correctly identifies both tissue-specific and housekeeping genes. Ranking and binning genes by entropy allowed us to begin to deconstruct core promoters into features directing when and where the gene will be expressed. We verified and extended previous observations [2] about the correlation of CpG islands with housekeeping genes and embryonic genes. We then identified differences in the base composition profile of promoters of tissue-specific and nonspecific genes. Next, we identified correlations between, on the one hand, the TATA box and tissue-specific genes, and on the other hand, the YY1 site and nonspecific genes. Finally, we identified trends in promoter classes based on CpG island and TATA box status and associated them with common cellular locations and biological processes. Similar observations were also observed for TATA box and Q-selected genes in pancreas.

The identification of an association between promoter type and cellular location and biological function, while an important step in a fundamental understanding of biology, also has practical significance, as the genes in the CGI-/TATA+ and CGI-/TATA- classes are enriched for tissue-specific extracellular and cell surface proteins. Such genes are likely to be useful drug targets. Thus entropy *H*_*g *_and *Q *have allowed us to discover fundamental properties of mammalian Pol II promoters and should allow serve to aid understanding of expression in particular tissues of interest.

The validity of our approach is supported by findings in other work and by the fact that they are robust with respect to the algorithm used to process the expression data. Our finding that most genes are regulated in a tissue-dependent manner is consistent with another analysis of gene expression [14], which found that housekeeping genes cluster in a tissue-specific manner. Thus, it appears, even the most basic biological functions are subject to regulation. The tissue trees we produced contain relationships similar to those in an analysis [48] of mid-specificity genes, including the close relation between lung, and the immune system-related organs spleen and thymus. That analysis is based on a different method and a different set of expression data gives us confidence that *Q*_*g*|*t *_is properly identifying genes that are specific to a tissue. The GNF-GEA expression data we analyzed was processed with the MAS4 [49] algorithm. We reanalyzed the data from this study after reprocessing it with the more recent Robust Multichip Average (RMA) algorithm [50]. This algorithm tends to suppress low-level signals and we found that most genes appeared to be more tissue specific, that is, had lower *H*, in the RMA-processed data compared to the reported values. Although this affects some of the precise values of numbers we have reported it does not alter any of the fundamental trends or results. We include tissue specificities based on both analyses in Additional data files 1 and 2.

Our analysis focused on only a few sequence features and although we found good correlations, two aspects of our results indicate that there are other regulatory mechanisms not yet identified. First, there is a gradual transition in the frequency of the TATA box and CpG islands between the most and least tissue-specific genes. Second, while these features are strong indicators of high and low specificity, they are far from perfect predictors. Indeed, the middle range of entropies contains a mix of all promoter classes in large numbers, indicating that it is possible to achieve tissue-specific expression with any promoter class. YY1 may be an example of such a supplementary mechanism. While occurring in only 16% of genes, it is very strictly confined to low-specificity genes and is a better indicator of low specificity than CpG islands. We expect that other such signals will be found.

Anatomical resolution is an issue with the datasets used in this study. For example, the pancreas consists of exocrine cells, ductal cells and islet cells of several types. The bulk pancreas was used to generate the GNF-GEA data, so the reported expression level is the average mRNA concentrations weighted by the cell-type count. This approximation reduces the maximum possible entropy and, more significantly, can make the apparent entropy different from the true entropy. Genes highly and specifically expressed in a cell type with a small population may currently appear to be ubiquitous with very low overall expression. Genes expressed in a few tissues may be revealed to be less tissue specific as more cell types are measured in detail. Genes that appear to be ubiquitously expressed may turn out to not to be expressed in a few cell types. It will be interesting to see whether data with higher anatomical resolution will help to increase the accuracy of the rules we have identified here for identifying tissue-specific and nonspecific promoters.

Our method can be also applied to other sources of expression data including SAGE, reverse transcription PCR (RT-PCR) and *in situ *hybridization data. SAGE has the advantage of sensitivity, as these studies generally sequence to much greater depths than EST libraries [51]. *In situ *hybridization data may increase the anatomical resolution of the data. Qualitative intensities, for example, '0', '+', or '+++', can be converted to representative numeric values as appropriate. Our method can also be applied to other collections of conditions beside normal tissues, for example, different types of cancers or samples of the same cancer from multiple patients. Modification of our method to account for temporal changes in tissue specificity represents another direction for future work.

The analysis presented here focuses on genes rather than on transcripts generated from different promoters from the same gene. The rate of the occurrence of alternative transcription start sites is at least 9% [52] and may be as high as 25% [53]. The promoters we used were specified by the DBTSS dataset but there may be alternative promoters with different characteristics and tissue-specific usage patterns. Analyses based on different RNA species can easily be incorporated into our approach and is an area of future research.

Our results for CpG island frequency in very tissue-specific genes are lower than recent reports [3] that were based upon present/absent calls, that is, tissue counting, using ESTs to measure tissue specificity. This may be due to two reasons. First, as we described in Results, a significant fraction of genes will show no evidence of expression in poorly sampled tissues. A poorly sampled nonspecific gene will appear therefore more tissue specific than it actually is and this increases the number of apparently tissue-specific genes with CpG islands. Second, when we use microarray data and determine tissue specificity by counting tissues expressing above the median value of 200 AU, we see (data not shown) rates of CpG island occurrence in 'specific' genes similar to those reported in [3]. Thus, we conclude that including the variation of expression levels rather than mere presence/absence is important for identifying very tissue-specific genes as assessed by start CpG islands.

These results present an initial look at the correlation between tissue specificity, CpG islands and binding sites for selected transcription factors that interact with the basal transcription apparatus. Using a novel approach with entropy-based metrics, we have begun to lay out the framework for promoter function by identifying strong correlations between tissue-specific or ubiquitous expression and a number of these sequence features. We plan to extend this work in several ways. First, we plan to identify correlations with other known transcription-factor-binding sites and novel motifs identified as over-represented in promoter regions [54]. Second, these results will help to understand regulation by combinations of multiple upstream transcription factors in genes specific to particular tissues or clusters of tissues.

## Conclusions

We have used Shannon entropy to quantify and rank the tissue specificity of genes using tissue-survey data. First, this has allowed us to assess the prevalence of tissue-specific regulation; we find that most genes show evidence of some degree of tissue-dependent variation in expression levels. It has also allowed us to find and evaluate associations between promoter features and tissue specificity. We have verified and extended understanding of known associations between, on the one hand, CpG islands and the least tissue-specific genes and, on the other hand, the TATA box and the most tissue-specific genes. However, they are not the sole determinants of tissue-specific expression, as indicated by mid-specificity genes that exhibit a mix of all promoter classes. The class of CGI-/TATA- promoters has emerged as the second most common class of promoter overall and the most common promoter class in mid-specificity genes. Therefore, additional determinants of tissue specificity remain to be found. We have identified one potential determinant, a downstream YY1 site, which is very strongly associated with the least tissue-specific genes but is a relatively rare feature of these promoters. Finally, we have also been able to associate trends in the localization and function of protein products of genes according to their promoter class. Many of the CGI-/TATA+ genes code for highly expressed, very tissue specific, extracellular proteins involved in a cell's response to the environment. CGI-/TATA- genes are also involved in response to the environment, but are found more uniformly across the spectrum of tissue specificity, are not as highly expressed as CGI-/TATA+ genes, and very often code for membrane-bound proteins. CGI+/TATA- genes are more likely to be located in the cytoplasm or nucleus and, as expected, carry out housekeeping functions. All of the results we report are found in both human and mouse and so may reflect general principles of all mammalian species.

## Materials and methods

### Processing GNF-GEA [22] and DoTS [33] data

The GNF-GEA data are processed as described [22]. Given a set of *N *tissues we define *p*_*t*|*g *_= *w*_*g*,*t*_/∑_1 ≤ *t *≤ *N*_*w*_*g*,*t *_where *w*_*t *_is the expression level of the gene *g *in tissue *t*. DoTS, available through the AllGenes [33] site, contains ESTs and mRNAs assembled into transcripts that are then clustered into genes. We did not consider any transcript that contains only one EST as this may represent a spurious sequence and did not consider any gene with fewer than five ESTs because they provide a poor estimate of H_*g*_. To accommodate the great disparity in sampling depth across tissues we normalized EST counts by tissue. To avoid artificially low entropies for genes that contain relatively few ESTs we used pseudocounts to smooth the data. The expression level of a gene in a tissue is computed as *w*_*g*,*t *_= (*n*_*g*,*t *_+ 1)/(N_*t *_+ *N*_*g*_) where *n*_*g*,*t *_is the number of ESTs from libraries for a tissue included in a gene, *N*_*t *_is the total number of ESTs from a tissue assembled into genes, and *N*_*g *_is the number of genes. We used different sets of tissues depending on the task. *H*_*g *_and *Q *measures in Figure 1 used the full GNF-GEA mouse set with a few modifications; adipose tissue and brown fat were merged, epidermis and snout epidermis were merged, digits and tongue were not considered as they are both a combination of skeletal muscle and epidermis. The expression level for a set of merged tissues is the median of the individual tissue replicate medians. For comparison of microarray and EST data we used a set of 27 tissues that were common to both datasets and merged the CNS and peripheral nervous system tissues.

### Estimating variance

To estimate the variance in *H *and Q, we took advantage of tissue replicates in the GNF-GEA data. Using the mouse dataset, we repeatedly sampled one of the measurements from each pair of replicates and computed *H *for each gene. We then computed the variance of the distribution of the estimates of *H *for each gene and show the survivor distribution function in Figure 2. The variance of *Q *was computed in a similar manner.

### Clustering tissues

Clustering was based on the *Q *scores for the set of mouse genes with *Q*_*g*|*t *_≤ 7 for at least one tissue and expressing at least 200 AU in at least one tissue in the GNF-GEA data. There were 1,786 Affymetrix probe sets selected. The tree in Figure 3 was built by sampling 5,000 sets of 1,000 probe sets and clustering tissues using Pearson correlation and a centered measure using the XCLUSTER [55] program. The consensus tree was built using the program CONSENSE in the PHYLIP [56] package with the default parameters.

### Identifying genes specific to a set of tissues

The total entropy of all tissues under a node can be computed at each node in the hierarchy using a generalization of the grouping theorem [57]. If the entropy of a gene at a node is close to the maximum possible entropy for the number of tissues under the node, then we select it and compute a *Q*_*g*,*n *_for the gene at the node. Using *Q*_*g*,*n *_we can rank genes by specificity to a cluster of tissues just as we can for an individual tissue.

### Predicting CpG islands

We predicted CpG islands using the program NEWCGREPORT in the EMBOSS [58] package with the default parameters which require a minimum length of 200 bp, C+G fraction of 0.6 and ratio of observed over expected CpG of 0.5.

### Statistical significance in embryonic expressed genes

We computed statistical significance of differences between all embryonic-expressed genes and adult-specific rates using a hypergeometric distribution. We start with a collection of *N *CGI+ genes, *n*_*e *_of which are expressed in the embryo, that is, marked as special. The *N*_*A *_tissue-specific genes in the adult are considered a random sample from the original *N *and we compute the probability of finding that at least (or at most) *n*_*ae *_of these were expressed in the embryo.

### Modeling distribution of entropy from uniform genes

To model the effect of experimental variability, we computed the distribution of the difference between expression levels of individual replicates for each gene and tissue and the mean expression level across replicates as a function of the mean expression level. This distribution was well fit by an exponential distribution with a parameter that depends on the mean expression level. Thus, given an 'ideal' expression level, we can estimate what the experimental variability will be. To model a uniformly expressed gene, we assume that a gene has some average expression level across all tissues and then allow the expression levels in individual tissues to follow a narrow distribution of random fold changes from that level. Specifically, we assumed that the log base 2 of the fold changes is distributed according to a normal distribution with mean equal to 0 and a standard deviation (*s*). The standard deviation can be adjusted to control the amount of biological variation a 'uniformly' expressed gene is allowed to show. For example, setting *s *= 0.5 means that about 68% of the fold changes between a particular tissue and the nominal level are within 1.4 up or down from the nominal level, that is, a twofold change from the lowest to the highest levels. Larger fold changes are expected to occur in 32% of tissues. This model allows significant variation and so is arguably close to the upper limit of variation allowable for a gene that shows no tissue specificity. We also used *s *= 0.25 as a more stringent definition of uniform expression. We sampled mean expression levels from the distribution of observed mean expression levels and sampled entropy values from the probability model. An entropy threshold was estimated by sampling approximately 5,000 random expression profiles and determining the value for a *p*-value of 0.002. This process was repeated 10 times and the corresponding thresholds and fraction of genes were computed. The thresholds spanned a range of less than 0.01 bit. The tissue-dependent gene fractions never varied by more than one percentage point in either direction.

### Statistical significance of co-occurrence

We estimated the statistical significance of the co-occurrence of motifs using the hypergeometric distribution. Given two motifs with occurrence counts *n*_1 _and *n*_2_, measured in the same set of *N *promoters, and a co-occurrence count of *n*_12_, we compute the significance as the probability of finding no more than (or at least) *n*_12 _hits in a random selection of *n*_2 _promoters from a pool of *N *promoters where *n*_1 _of them are 'special'.

### Comparison of frequency on independent sets

Given two sets of size *N*_1 _and *N*_2 _and positive observations *n*_1 _and *n*_2 _in each, we computed the probability that the underlying rates are different using an exact calculation of the binomial distribution to compute the probability of seeing at least (or no more) than *n*_*i *_matches in *N*_*i *_trials where the rate is assumed to be *r *= *n*_*j*_/*N*_*j*_. We estimated *r *using the larger of the two sets.

### Two binomial populations

We used the normal approximation to the difference of the proportions normalized by their variance to compute a *z*-score.

### Promoter sequences

We obtained promoter sequence in two ways. The *H*-based set of analyses used links from Affymetrix probe sets to RefSeq identifiers to select alignments from the DBTSS promoter sequences covering the (-1000, 200) region downloaded from the DBTSS website [59]. The *Q*-based analyses of TATA box and initiator elements used genomic locations of DoTS genes on UCSC Golden Path release mm3 [60,61] to identify gene names. Promoter sequences consisting of the 350 bp of the upstream region were then extracted from Ensembl [62]. The mouse homologs were also used as annotated in Ensembl.

### Core motifs

The *H*-based analysis used core promoter element models from EPD [36,63]. The fraction of promoters containing each matrix was determined as follows for each set of genes (with and without CpG islands in each entropy bin) individually. Having verified that the positional distribution of each motif was sharply peaked at the appropriate place in the promoter sequences ((-40, -20) region for TATA and (-20, +20) region for the initiator element) we considered only the predictions in these windows from all genes. We used the log-likelihood function to score each subsequence against each matrix using the published score cut-offs. The YY1 motif was found in essentially every run of AlignACE and MEME performed on the downstream regions of ubiquitous CGI+ promoters. We explored different motif widths and other settings and selected version that achieved a combination of good coverage and conservation. In all cases we estimated the background rate of random occurrence of motifs by repeatedly scrambling the individual sequences over a 10 bp window to create approximately 1,000 test sequences for each combination of CpG island status and specificity range. These sequences were scored in the same manner as the unscrambled sequences. We estimated the statistical significance of differences of observed frequencies using exact computation of the binomial distribution. The *Q*-based analyses of core motifs used the TATA box motif (TATAA) and initiator element (YYANWYY). Motif searches were carried out using the tool patternmatch from the biological workbench 3.2 [64]. Only the TATAA instance located closest to the start of the mRNA's alignment to the genome was used. Matches to the initiator element were required to be downstream of the TATAA box when present.

### YY1 motif

We used an AlignACE-derived weight matrix (shown in Figure 6a) to assess the occurrence of YY1-like sites as it contained the YY1 consensus and was built using approximately 100 sites which is many more than previously published weight matrices [43,65] also shown in Figure 6a.

### GO association analysis

We submitted Affymetrix probe set ids of interest to the DAVID website [45,46] and compared them either to all probe sets on the appropriate Affymetrix chips or to all genes in the selected entropy range. We compensated for multiple testing by requiring the reported *p*-values be better than either 0.05/1472 = 0.00003 (cellular component) or 0.05/8972 = 0.000006 (biological process) using the number of GO terms for the corresponding GO divisions in a Bonferroni correction.

### RMA quantification

We obtained CEL files for the GNF-GEA study from and re-quantified them using the gcrma package [66] in the Bioconductor [67] project for the R statistical analysis program [68]. We use the gcrma options 'type=c('fullmodel')' and 'fast=T'.

## Additional data files

Two additional data files are available with the online version of this article. They contain H and Q values for all normal tissues in the GNF-GEA data set for both human (Additional data file 1) and mouse (Additional data file 2) using both the MAS4 and RMA quantification methods. The RMA data were normalized to yield a common median of 3.75 (human) and 3.22 (mouse) prior to the H and Q calculation. The files are in Excel format. The data for each tissue are placed in separate worksheets. Each worksheet contains H- and Q-values, the expression value of the gene in the worksheet's tissue, and its maximum expression across all tissues in the file, the gene symbol, RefSeq, SwissProt, and Unigene ID, and a description. The rows in each worksheet are sorted by increasing values of Q using the RMA data. Thus the top of each worksheet displays the genes most specific to that worksheet's tissue.

## Supplementary Material

Additional File 1 A table showing H and Q values for all normal human tissues in the GNF-GEA dataset. H and Q values for all normal tissues in the GNF-GEA dataset for human using both the original MAS4 quantification and our RMA re-quantification. The RMA data were normalized to yield common medians of 3.75 prior to the H and Q calculation. The data for each tissue are placed in separate worksheets. Each worksheet contains H- and Q-values, the expression value of the gene in the worksheet's tissue, and its maximum expression across all tissues in the file, the gene symbol, RefSeq, SwissProt, and Unigene ID, and a description. The rows in each worksheet are sorted by increasing values of Q using the RMA data. Thus the top of each worksheet displays the genes most specific to that worksheet's tissue. Click here for file

Additional File 2 A table showing H and Q values for all normal mouse tissues in the GNF-GEA dataset. H and Q values for all normal tissues in the GNF-GEA dataset for mouse using both the original MAS4 quantification and our RMA re-quantification. The RMA data were normalized to yield common medians of 3.22 prior to the H and Q calculation. The data for each tissue are placed in separate worksheets. Each worksheet contains H- and Q-values, the expression value of the gene in the worksheet's tissue, and its maximum expression across all tissues in the file, the gene symbol, RefSeq, SwissProt, and Unigene ID, and a description. The rows in each worksheet are sorted by increasing values of Q using the RMA data. Thus the top of each worksheet displays the genes most specific to that worksheet's tissue. Click here for file

## Figures and Tables

**Table 1:** The top five most tissue-specific genes for representative tissues

Tissue	Probe set ID	*H*	*Q*	RefSeq	Description
Amygdala	96055_at	3.2	5.8	NM_031161	Cholecystokinin
	93178_at	2.7	5.8	NM_019867	Neuronal guanine nucleotide exchange factor
	93273_at	3.7	5.8	NM_009221	Synuclein, alpha
	92943_at	3.5	6.0	NM_008165	Glutamate receptor, ionotropic, AMPA1 (alpha 1)
	95436_at	3.3	6.1	NM_009215	Somatostatin
					
Lymph node	98406_at	2.7	4.0	NM_013653	Chemokine (C-C motif) ligand 5
	98063_at	1.6	4.1	-	Glycosylation dependent cell adhesion molecule 1
	99446_at	2.5	4.1	NM_007641	Membrane-spanning 4-domains, subfamily A, member 1
	92741_g_at	3.3	4.5	-	Immunoglobulin heavy chain 4 (serum IgG1)
	102940_at	2.8	4.6	NM_008518	Lymphotoxin B
					
Liver	94777_at	1.3	2.1	-	Albumin 1
	101287_s_at	1.6	2.2	NM_010005	Cytochrome P450, 2d10
	99269_g_at	1.5	2.2	NM_019911	Tryptophan 2,3-dioxygenase
	100329_at	1.4	2.3	NM_009246	Serine protease inhibitor 1-4
	94318_at	1.6	2.3	NM_013475	Apolipoprotein H

Genes must express at 200 AU in one or more tissues. A full list of all genes is available in the Additional data files 1 and 2.

**Table 2:** The list of tissues used in this study

GNF+GEA tissues	Comparison to EST	Hierarchical clustering
DRG	PNS	Nervous system
Trigeminal	CNS	
Hippocampus	CNS	
Amygdala	CNS	
Frontal_cortex	CNS	
Cortex	CNS	
Striatum	CNS	
Olfactory_bulb	CNS	
Hypothalamus	CNS	
Spinal_cord_lower	CNS	
Spinal_cord_upper	CNS	
Cerebellum	CNS	
Eye	Eye	
		
Spleen	Spleen	Immune System + trachea
Lymph_node	Lymph_node	
Trachea	Trachea	
Thymus	Thymus	
Bone_marrow	Bone	
Bone	Bone	
Lung	Lung	
		
Uterus	Uterus	Reproductive organs
Umbilical cord	Umbilical_cord	
Placenta	Plancenta	
Ovary	Ovary	
		
Epidermis, snout_epidermis	Epidermis	
		
Heart	Heart	Muscle
Skeletal_muscle	Skeletal_muscle	
		
Adipose_tissue, brown_fat	Fat	
Adrenal_gland	Adrenal_gland	
		
Stomach	Stomach	Digestive tract
Bladder	Bladder	
Small_intestine	Small_intestine	
Large_intestine	Large_intestine	
		
Gall bladder	Gall_bladder	Gall bladder, liver, and kidney
Liver	Liver	
Kidney	Kidney	
		
Salivary_gland	Salivary_gland	
Thyroid	Thyroid	
Mammary_gland	Mammary_gland	
Prostate	Prostate	
Testis	Testis	
Tongue	Tongue	
Digits	Digits	

The list of tissues available in the mouse GNF+GEA survey, groupings of tissues used to compare microarray and EST-based entropy estimates, and tissue groups discovered by clustering tissues on the basis of genes expressed in common.

**Table 3:** The top five most group-specific mouse genes for selected tissue groups

Tissue cluster	Probe Set ID	*H*	*Q*	RefSeq	Description
Nervous system	100047_at	3.3	3.4	NM_011428	Synaptosomal-associated protein, 25 kDa
	103030_at	3.5	3.6		Dynamin
	97983_s_at	3.7	3.8	NM_009295	Syntaxin binding protein 1
	98339_at	3.7	3.8	NM_018804	Synaptotagmin 11
	94545_at	3.7	3.8	NM_153457	Reticulon 1
					
Immune system	96648_at	2.807	2.882	NM_009898	Coronin, actin binding protein 1a
	93584_at	3.373	3.622		Immunoglobulin heavy chain 6 (heavy chain of IgM)
					
	101048_at	3.541	3.876	NM_011210	Protein tyrosine phosphatase, receptor type, C
	94278_at	3.495	3.923	NM_008879	Lymphocyte cytosolic protein 1
	100156_at	3.609	4.039	NM_008566	Mini chromosome maintenance deficient 5
					
Liver and gall bladder	94777_at	1.280	1.326		Albumin 1
	100329_at	1.394	1.464	NM_009246	Serine protease inhibitor 1-4
	99269_g_at	1.471	1.561	NM_019911	Tryptophan 2,3-dioxygenase
	99862_at	1.503	1.595	NM_013465	Alpha-2-HS-glycoprotein
	96846_at	1.515	1.607	NM_080844	Serine (or cysteine) proteinase inhibitor, clade C (antithrombin), member 1

The tissue groups were identified in a consensus clustering of tissues based on common tissue-specific genes. The *Q *value is for the gene and tissue group. To ensure uniform expression across the tissue group, genes were required to have an entropy on the tissue group that was 90% of the maximum possible for the group.

**Table 4:** CpG islands are correlated with embryonic expression even for tissue-specific genes

Gene type	CpG island state	Total genes considered	Expressed genes	Fraction	Fraction ratio
Embryo	CGI+	933	365	39%	2.8
	CGI-	1007	139	14%	

Adult-specific	CGI+	29	8	29%	4
	CGI-	180	12	7%	

We determined the fraction of genes with (39%) and without (14%) start CpG islands that are expressed in the early embryo. A gene is 2.8 (= 0.39/0.14) times more likely to be expressed in the early embryo if it has a start CpG island. If we then consider genes that go on to be specific in the adult, we find the ratio of CGI+/CGI- genes is now 4 = 0.28/0.07. The differences in rates between CpG island status within each stage are significant (*P *< 0.0005; binomial). Of the between-stage comparisons, only the CGI- adult-specific/embryo change is significant (*P *= 0.0009; hypergeometric).

**Table 5:** The most significant indicators of the degree of tissue specificity: start CpG island, TATA box, and YY1 site

Features			Total fraction	*H *0-3	*H *3-4	*H *4-5
		
CGI	TATA	YY1		Most specific	Semi-specific	Least specific
			**3,552**	**271**	**602**	**2679**
			1.00	0.08	0.17	0.75
						
CGI+			**2,434**	**56**	**306**	**2072**
			0.69	0.02	0.13	0.85
				0.30	0.74	1.13
CGI-			**1,118**	**215**	**296**	**607**
			0.31	0.19	0.26	0.54
				2.52	1.56	0.72
						
	TATA+		**604**	**136**	**175**	**293**
			0.17	0.23	0.29	0.49
				2.95	1.71	0.64
	TATA-		**2,949**	**135**	**427**	**2,387**
			0.83	0.05	0.14	0.81
				0.60	0.85	1.07
						
CGI+	TATA+		**284**	**19**	**82**	**183**
			0.08	0.07	0.29	0.64
				0.88	1.70	0.85
CGI-	TATA+		**320**	**117**	**93**	**110**
			0.09	0.37	0.29	0.34
				4.79	1.71	0.46
CGI+	TATA-		**2,150**	**37**	**224**	**1,889**
			0.61	0.02	0.10	0.88
				0.23	0.61	1.16
CGI-	TATA-		**798**	**98**	**203**	**497**
			0.22	0.12	0.25	0.62
				1.61	1.50	0.83
						
		YY1+	**293**	**1**	**16**	**276**
			0.08	0.00	0.05	0.94
				0.04	0.32	1.25
						
CGI+		YY1+	**261**	**1**	**10**	**250**
			0.07	0.00	0.04	0.96
				0.05	0.23	1.27
CGI+		YY1-	**2,173**	**55**	**296**	**1,822**
			0.61	0.03	0.14	0.84
				0.33	0.80	1.11
CGI-		YY1-	**1,086**	**215**	**290**	**581**
			0.31	0.20	0.27	0.53
				2.59	1.58	0.71
CGI-		YY1+	**32**	**0**	**6**	**26**
			0.01	0.00	0.19	0.81
				0.00	1.11	1.08

The three columns on the left indicate the combination of features considered; empty cells indicate that the feature is not considered. The 'Total fraction' column indicates the number of promoters with each feature combination (in bold) and the corresponding fraction of all genes considered. The three columns on the right give statistics for matching genes in three bands of tissue specificity. The top two lines give the number and corresponding fraction of all genes considered for each band. For each feature combination, the numbers indicate the number (top, bold), fraction (middle), and enrichment ratio (bottom) of matching genes. The enrichment ratio is the fraction of promoters of genes in the entropy band that contain a feature divided by the band's fraction among all genes considered. For example, specific genes are best recognized by a combination of TATA box (TATA+) and lack of a CpG island (CGI-), which enriches the fraction of such genes from 8% to 37% - a factor of 4.79. Nonspecific genes are most specifically recognized by CpG islands and YY1 sites, which returns a set that is 96% nonspecific genes, but only matches 7%/75% = 10% of the nonspecific genes.

**Table 6:** Over-represented Gene Ontology (GO) terms for cellular component and biological process of genes by promoter class

	Cellular component/biological process	Human only	Mouse only
CGI-/TATA+	Extracellular, extracellular space	**-**	Intermediate filament (cytoskeleton)
	Response to stimulus	Cell-cell signaling, organismal physiological process, inflammatory response, innate immune response, response to pest/pathogen/parasite	-
			
CGI+/TATA-	Cell, cytoplasm, intracellular, mitochondrion	Nucleus, ribonucleoprotein complex	**-**
	**-**	Nucleobase, nucleoside, nucleotide and nucleic acid metabolism, intracellular transport, metabolism, protein transport, intracellular protein transport, RNA processing, RNA metabolism, cell cycle, mitotic cell cycle	**-**
			
CGI-/TATA-	(Integral to) (plasma) membrane	-	Extracellular, extracellular space
	Organismal physiological process, defense response, immune response, response to biotic stimulus, response to stimulus, response to external stimulus	Response to pest/pathogen/parasite, cell communication, response to wounding, cellular defense response, signal transduction	Complement activation, complement activation (classical pathway), humoral defense mechanism (*sensu *Vertebrata), humoral immune response

All terms were selected using a *p*-value ≤ 0.05 (corrected for multiple testing). Terms common to human and mouse are listed in the second column. The two columns on the right indicate any additional terms found in only one species. The CGI-/TATA+ terms are consistent with a model of strong condition-specific induction, CGI+/TATA- terms are consistent with housekeeping functions. CGI-/TATA- terms indicate support for cell sensing and communication functions. No significant results were found for CGI+/TATA+ genes.

## References

[B1] Bird AP (1984). DNA methylation - how important in gene control?. Nature.

[B2] Bird AP (1984). DNA methylation versus gene expression.. J Embryol Exp Morphol.

[B3] Ponger L, Duret L, Mouchiroud D (2001). Determinants of CpG islands: expression in early embryo and isochore structure.. Genome Res.

[B4] Smale ST, Baltimore D (1989). The 'initiator' as a transcription control element.. Cell.

[B5] Shi Y, Seto E, Chang LS, Shenk T (1991). Transcriptional repression by YY1, a human GLI-Kruppel-related protein, and relief of repression by adenovirus E1A protein.. Cell.

[B6] Seto E, Shi Y, Shenk T (1991). YY1 is an initiator sequence-binding protein that directs and activates transcription in vitro.. Nature.

[B7] Riggs KJ, Saleque S, Wong KK, Merrell KT, Lee JS, Shi Y, Calame K (1993). Yin-yang 1 activates the c-myc promoter.. Mol Cell Biol.

[B8] Riggs KJ, Merrell KT, Wilson G, Calame K (1991). Common factor 1 is a transcriptional activator which binds in the c-myc promoter, the skeletal alpha-actin promoter, and the immunoglobulin heavy-chain enhancer.. Mol Cell Biol.

[B9] Wasserman WW, Fickett JW (1998). Identification of regulatory regions which confer muscle-specific gene expression.. J Mol Biol.

[B10] Krivan W, Wasserman WW (2001). A predictive model for regulatory sequences directing liver-specific transcription.. Genome Res.

[B11] Ringrose L, Rehmsmeier M, Dura JM, Paro R (2003). Genome-wide prediction of Polycomb/Trithorax response elements in *Drosophila melanogaster*.. Dev Cell.

[B12] Berman BP, Pfeiffer BD, Laverty TR, Salzberg SL, Rubin GM, Eisen MB, Celniker SE (2004). Computational identification of developmental enhancers: conservation and function of transcription factor binding-site clusters in *Drosophila melanogaster *and *Drosophila pseudoobscura*.. Genome Biol.

[B13] Wasserman WW, Sandelin A (2004). Applied bioinformatics for the identification of regulatory elements.. Nat Rev Genet.

[B14] Hsiao LL, Dangond F, Yoshida T, Hong R, Jensen RV, Misra J, Dillon W, Lee KF, Clark KE, Haverty P (2001). A compendium of gene expression in normal human tissues.. Physiol Genomics.

[B15] Arbeitman MN, Furlong EE, Imam F, Johnson E, Null BH, Baker BS, Krasnow MA, Scott MP, Davis RW, White KP (2002). Gene expression during the life cycle of Drosophila melanogaster.. Science.

[B16] Waterston RH, Lindblad-Toh K, Birney E, Rogers J, Abril JF, Agarwal P, Agarwala R, Ainscough R, Alexandersson M, An P (2002). Initial sequencing and comparative analysis of the mouse genome.. Nature.

[B17] Suzuki Y, Yamashita R, Sugano S, Nakai K (2004). DBTSS, DataBase of Transcriptional Start Sites: progress report 2004.. Nucleic Acids Res.

[B18] Carninci P, Waki K, Shiraki T, Konno H, Shibata K, Itoh M, Aizawa K, Arakawa T, Ishii Y, Sasaki D (2003). Targeting a complex transcriptome: the construction of the mouse full-length cDNA encyclopedia.. Genome Res.

[B19] Strausberg RL, Feingold EA, Klausner RD, Collins FS (1999). The mammalian gene collection.. Science.

[B20] Gitton Y, Dahmane N, Baik S, Ruiz i, Altaba A, Neidhardt L, Scholze M, Herrmann BG, Kahlem P, Benkahla A, Schrinner S (2002). A gene expression map of human chromosome 21 orthologues in the mouse.. Nature.

[B21] Reymond A, Marigo V, Yaylaoglu MB, Leoni A, Ucla C, Scamuffa N, Caccioppoli C, Dermitzakis ET, Lyle R, Banfi S (2002). Human chromosome 21 gene expression atlas in the mouse.. Nature.

[B22] Su AI, Cooke MP, Ching KA, Hakak Y, Walker JR, Wiltshire T, Orth AP, Vega RG, Sapinoso LM, Moqrich A (2002). Large-scale analysis of the human and mouse transcriptomes.. Proc Natl Acad Sci USA.

[B23] Safran M, Chalifa-Caspi V, Shmueli O, Olender T, Lapidot M, Rosen N, Shmoish M, Peter Y, Glusman G, Feldmesser E (2003). Human gene-centric databases at the Weizmann Institute of Science: GeneCards, UDB, CroW 21 and HORDE.. Nucleic Acids Res.

[B24] Hayashizaki Y (2003). RIKEN mouse genome encyclopedia.. Mech Ageing Dev.

[B25] Wheeler DL, Church DM, Federhen S, Lash AE, Madden TL, Pontius JU, Schuler GD, Schriml LM, Sequeira E, Tatusova TA (2003). Database resources of the National Center for Biotechnology.. Nucleic Acids Res.

[B26] Boguski MS, Lowe TM, Tolstoshev CM (1993). dbEST - database for 'expressed sequence tags'.. Nat Genet.

[B27] Huminiecki L, Lloyd AT, Wolfe KH (2003). Congruence of tissue expression profiles from Gene Expression Atlas, SAGEmap and TissueInfo databases.. BMC Genomics.

[B28] Vinogradov AE (2003). Isochores and tissue-specificity.. Nucleic Acids Res.

[B29] Stanton JA, Macgregor AB, Green DP (2003). Identifying tissue-enriched gene expression in mouse tissues using the NIH UniGene database.. Appl Bioinformatics.

[B30] Fuhrman S, Cunningham MJ, Wen X, Zweiger G, Seilhamer JJ, Somogyi R (2000). The application of shannon entropy in the identification of putative drug targets.. Biosystems.

[B31] Cunningham MJ, Liang S, Fuhrman S, Seilhamer JJ, Somogyi R (2000). Gene expression microarray data analysis for toxicology profiling.. Ann NY Acad Sci.

[B32] Peterson LE (2002). CLUSFAVOR 5.0: hierarchical cluster and principal-component analysis of microarray-based transcriptional profiles.. Genome Biol.

[B33] DoTS. http://www.allgenes.org.

[B34] Shannon C (1949). The Mathematical Theory of Communication.

[B35] Eisenberg E, Levanon EY (2003). Human housekeeping genes are compact.. Trends Genet.

[B36] Bucher P (1990). Weight matrix descriptions of four eukaryotic RNA polymerase II promoter elements derived from 502 unrelated promoter sequences.. J Mol Biol.

[B37] Cook T, Gebelein B, Urrutia R (1999). Sp1 and its likes: biochemical and functional predictions for a growing family of zinc finger transcription factors.. Ann NY Acad Sci.

[B38] Li L, He S, Sun JM, Davie JR (2004). Gene regulation by Sp1 and Sp3.. Biochem Cell Biol.

[B39] Lee JS, Galvin KM, Shi Y (1993). Evidence for physical interaction between the zinc-finger transcription factors YY1 and Sp1.. Proc Natl Acad Sci USA.

[B40] Natesan S, Gilman MZ (1993). DNA bending and orientation-dependent function of YY1 in the c-*fos *promoter.. Genes Dev.

[B41] McGuire AM, Hughes JD, Church GM (2000). Conservation of DNA regulatory motifs and discovery of new motifs in microbial genomes.. Genome Res.

[B42] Bailey TL, Baker ME, Elkan CP (1997). An artificial intelligence approach to motif discovery in protein sequences: application to steroid dehydrogenases.. J Steroid Biochem Mol Biol.

[B43] Shrivastava A, Calame K (1994). An analysis of genes regulated by the multi-functional transcriptional regulator Yin Yang-1.. Nucleic Acids Res.

[B44] Smale ST (1997). Transcription initiation from TATA-less promoters within eukaryotic protein-coding genes.. Biochim Biophys Acta.

[B45] Hosack DA, Dennis G, Sherman BT, Lane HC, Lempicki RA (2003). Identifying biological themes within lists of genes with EASE.. Genome Biol.

[B46] Dennis G, Sherman BT, Hosack DA, Yang J, Gao W, Lane HC, Lempicki RA (2003). DAVID: Database for Annotation, Visualization, and Integrated Discovery.. Genome Biol.

[B47] Harris MA, Clark J, Ireland A, Lomax J, Ashburner M, Foulger R, Eilbeck K, Lewis S, Marshall B, Mungall C (2004). The Gene Ontology (GO) database and informatics resource.. Nucleic Acids Res.

[B48] Yanai I, Benjamin H, Shmoish M, Chalifa-Caspi V, Shklar M, Ophir R, Bar-Even A, Horn-Saban S, Safran M, Domany E (2004). Genome-wide midrange transcription profiles reveal expression level relationships in human tissue specification.. Bioinformatics.

[B49] Lockhart DJ, Dong H, Byrne MC, Folletti MT, Gallo MV, Chee MS, Mittmann M, Wang C, Kobayashi M, Horton H, Brown EL (1996). Expression monitoring by hybridization to high-density oligonucleotide arrays.. Nat Biotechnol.

[B50] Bolstad BM, Irizarry RA, Astrand M, Speed TP (2003). A comparison of normalization methods for high density oligonucleotide array data based on variance and bias.. Bioinformatics.

[B51] Boheler KR, Stern MD (2003). The new role of SAGE in gene discovery.. Trends Biotechnol.

[B52] Zavolan M, van Nimwegen E, Gaasterland T (2002). Splice variation in mouse full-length cDNAs identified by mapping to the mouse genome.. Genome Res.

[B53] Zavolan M, Kondo S, Schonbach C, Adachi J, Hume DA, Hayashizaki Y, Gaasterland T (2003). Impact of alternative initiation, splicing, and termination on the diversity of the mRNA transcripts encoded by the mouse transcriptome.. Genome Res.

[B54] Marino-Ramirez L, Spouge JL, Kanga GC, Landsman D (2004). Statistical analysis of over-represented words in human promoter sequences.. Nucleic Acids Res.

[B55] Sherlock G (2001). Analysis of large-scale gene expression data.. Brief Bioinform.

[B56] Felsenstein J (1993). PHYLIP.. Seattle.

[B57] Ash RB (1965). Information Theory.

[B58] Rice P, Longden I, Bleasby A (2000). EMBOSS: the European Molecular Biology Open Software Suite.. Trends Genet.

[B59] Database of Transcriptional Start Sites. http://dbtss.hgc.jp/index.html.

[B60] Karolchik D, Hinrichs AS, Furey TS, Roskin KM, Sugnet CW, Haussler D, Kent WJ (2004). The UCSC Table Browser data retrieval tool.. Nucleic Acids Res.

[B61] Kent WJ, Sugnet CW, Furey TS, Roskin KM, Pringle TH, Zahler AM, Haussler D (2002). The human genome browser at UCSC.. Genome Res.

[B62] Birney E, Andrews D, Bevan P, Caccamo M, Cameron G, Chen Y, Clarke L, Coates G, Cox T, Cuff J (2004). Ensembl 2004.. Nucleic Acids Res.

[B63] Perier RC, Praz V, Junier T, Bonnard C, Bucher P (2000). The eukaryotic promoter database (EPD).. Nucleic Acids Res.

[B64] Subramaniam S (1998). The Biology Workbench - a seamless database and analysis environment for the biologist.. Proteins.

[B65] Matys V, Fricke E, Geffers R, Gossling E, Haubrock M, Hehl R, Hornischer K, Karas D, Kel AE, Kel-Margoulis OV (2003). TRANSFAC: transcriptional regulation, from patterns to profiles.. Nucleic Acids Res.

[B66] Wu Z, Irizarry R (2004). gcrma..

[B67] Gentleman RC, Carey VJ, Bates DM, Bolstad B, Dettling M, Dudoit S, Ellis B, Gautier L, Ge Y, Gentry J (2004). Bioconductor: open software development for computational biology and bioinformatics.. Genome Biol.

[B68] Team RDC (2004). R: A language and environment for statistical computing..

[B69] Schneider TD, Stephens RM (1990). Sequence logos: a new way to display consensus sequences.. Nucleic Acids Res.

